# Diffuse Gastric Cancer: A Summary of Analogous Contributing Factors for Its Molecular Pathogenicity

**DOI:** 10.3390/ijms19082424

**Published:** 2018-08-16

**Authors:** Shamshul Ansari, Boldbaatar Gantuya, Vo Phuoc Tuan, Yoshio Yamaoka

**Affiliations:** 1 Department of Environmental and Preventive Medicine, Oita University Faculty of Medicine, Yufu-City, Oita 879-5593, Japan; shamshulansari483@yahoo.com (S.A.); medication_bg@yahoo.com (B.G.); vophuoctuandr@gmail.com (V.P.T.); 2 Department of Internal Medicine, Gastroenterology unit, Mongolian National University of Medical Sciences, Ulaanbaatar-14210, Mongolia; 3 Department of Endoscopy, Cho Ray Hospital, Ho Chi Minh, Vietnam; 4 Department of Medicine, Gastroenterology and Hepatology Section, Baylor College of Medicine, Houston, TX 77030, USA

**Keywords:** gastric cancer, diffuse gastric cancer, hereditary diffuse gastric cancer, contributing factors

## Abstract

Gastric cancer is the third leading cause of cancer-related deaths and ranks as the fifth most common cancer worldwide. Incidence and mortality differ depending on the geographical region and gastric cancer ranks first in East Asian countries. Although genetic factors, gastric environment, and *Helicobacter pylori* infection have been associated with the pathogenicity and development of intestinal-type gastric cancer that follows the Correa’s cascade, the pathogenicity of diffuse-type gastric cancer remains mostly unknown and undefined. However, genetic abnormalities in the cell adherence factors, such as E-cadherin and cellular activities that cause impaired cell integrity and physiology, have been documented as contributing factors. In recent years, *H. pylori* infection has been also associated with the development of diffuse-type gastric cancer. Therefore, in this report, we discuss the host factors as well as the bacterial factors that have been reported as associated factors contributing to the development of diffuse-type gastric cancer.

## 1. Introduction

Gastric cancer (GC) was the most common cancer as of 1975 [1] and because of the lack of sophisticated advancements, most of the GC cases were diagnosed at the advanced stages with poor prognosis [2]. However, relying on the development of advanced endoscopic techniques and national policy on *Helicobacter pylori* eradication, currently GC can be detected at earlier stages and better interventions can be provided to prevent its advance in some countries, such as Japan and Korea [3,4]. In fact, the declining trend in the global incidence and mortality of GC has been observed over past decades; however, it still ranks as the fifth most common cancer and the third leading cause of cancer-related mortality worldwide with an estimated number of 841,000 deaths, including 530,000 deaths and 11.7 million disability-adjusted life years (DALYs) for men in 2013 [5]. Hence, GC is still a significant public health issue and is still an area of focus for many international organizations in terms of both the prevention and control of the disease. The incidence and mortality of GC varies according to geographical region and it remains the highest in East Asian countries in comparison with other parts of the world [1].

GC is a multifactorial, morphologically heterogeneous disease where adenocarcinoma accounts for almost 90% of cases and lymphoma up to 5% [6,7]. Histologically, the adenocarcinomas originate from the glandular epithelium of gastric mucosa, whereas almost 90% of the primary gastrointestinal lymphomas are of B cell lineage with few T-cell or Hodgkin lymphomas [6,8]. In general, most GCs are sporadic (90%) and a positive family history exists in approximately 10% of cases, of which 1–3% are hereditary [9,10]. Based on differences in morphology, epidemiology, pathology, and genetic profiles, adenocarcinoma is classified as the well-differentiated or intestinal type gastric cancer (IGC) accounting for 60% of cases that typically show cohesive groups of tumor cells with a well-defined glandular architecture leading to expanding growth pattern. Poorly-differentiated or diffuse type gastric cancer (DGC) accounts for 30% of cases; DGC lacks the intercellular adhesion, often observed with scattered signet-ring cell morphology predisposed to the diffuse invasion growth pattern throughout the stroma [11,12]. IGC is found in older patients and is associated more with environmental factors, such as high salty diet, smoking, obesity, and alcohol consumption [13,14,15], as well as *H. pylori* infection [16]. DGC is more commonly observed in younger patients [17,18]. IGC is the more common variant and its carcinogenic pathway is mainly caused by *H. pylori* infection, which predisposes a person to chronic gastritis, followed by atrophic gastritis, intestinal metaplasia, dysplasia, and finally carcinoma through the Correa’s cascade [19]. The latter three lesions—atrophic gastritis, intestinal metaplasia, and dysplasia—are considered precancerous lesions. IGC accounts for the vast majority of GC. Although the pathogenicity of IGC has been well-characterized and studied, that of DGC mostly remains undefined and is considered to be primarily genetically determined and less associated with environmental factors and the inflammatory cascade. Even though DGC accounts for a lower proportion, an increasing incidence of DGC has been reported [20]. Moreover, a minor proportion of DGC (1–3%) has been inherently linked and associated with germline alterations in cellular physiology, which is known as hereditary-DGC (HDGC) [21,22,23]. Along with the worse prognosis characterized by early age of onset, rapid disease progression, being highly metastatic, inherited possibility within family in comparison associated with IGC, DGC has become a challenge for researchers and physicians. In practice, due to the clinical importance, several guidelines about diagnosis criteria, treatment, and monitoring of hereditary DGC (HDGC) were established and updated by a multidisciplinary group including clinical geneticists, gastroenterologists, surgeons, oncologists, pathologists, molecular biologists, and dieticians [24,25,26]. Nonetheless, the underlying molecular pathways of the disease have not yet been well-studied and understood. Notably, a report summarizing the molecular pathogenicity of GC in general has been published previously [27]. However, in this report, we summarized the current understanding of published knowledge to create a possible outline of the contributing factors involved in the molecular pathogenicity of DGC in order to gain deeper awareness about its mechanism (Table 1).

## 2. Factors Associated with Molecular Pathogenicity of DGC

### 2.1. Role of E-Cadherin

**Figure 1:**
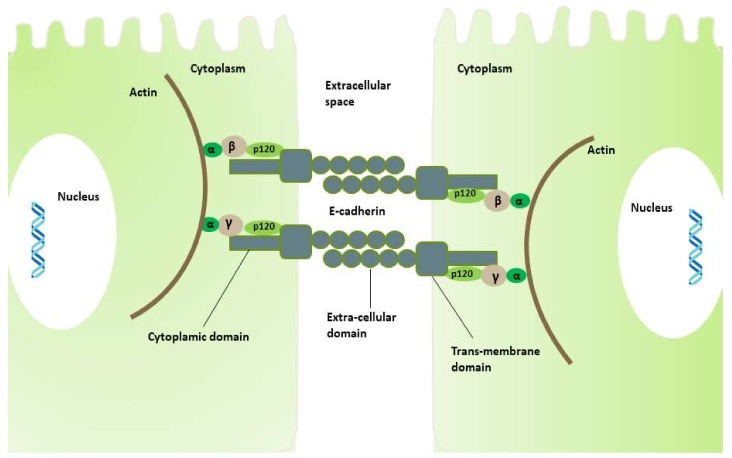
Cell–cell adhesion through E-cadherin. The extracellular domain of E-cadherin from adjacent cells is involved in cell adhesion and tight junction. The cytoplasmic domain forming a protein complex with catenins (α-, β-, and p120-) regulates the cytoskeleton protein and actin, which is an important protein for normal cell integrity.

For the normal maintenance of tissue morphogenesis and homeostasis, cell–cell adhesion is a critical phenomenon, also important for other cellular processes such as cell differentiation, cell survival, and cell migration, which are controlled by gene expression and signaling pathway activation [57]. E-cadherin (calcium-dependent classical cadherin), a trans-membrane glycoprotein consisting of three domains—extracellular, trans-membrane, and cytoplasmic—is involved in the cell–cell adhesion and tight adherent junctions that define cell differentiation and proliferation specificity of epithelial cells and invasion suppression [30,58,59,60]. The cytoplasmic domain of E-cadherin forms a protein complex with β- or γ-, p120-, and α-catenins linking the domain with the actin-myosin network that co-ordinates the specificity of cell shape, polarity, and function of the epithelial cells [61,62]. The extra-cellular domain of E-cadherin from the adjacent cells is involved in the cell adherence, providing a tight junction between the cells (Figure 1).

The glycoprotein E-cadherin is encoded by the cadherin (*CDH1*) gene, which is located in chromosome 16q22.1 and contains 16 exons with a 4.5-kb mRNA [63]. E-cadherin is one of the major tumor suppressors in GC and the structural modifications in its encoding gene *CDH1* or alterations in its expression have been found as the common events that suppress the broad-ranging functions of E-cadherin during cancer progression and contribute to the morphogenetic effects in cancer [10,28,61]. The common mutations in *CDH1* are the well-known mechanism for its deregulation [29,64]. According to the human gene mutation database (HGMD), 121 variants have been reported for *CDH1* alterations to date [65,66]. In addition to the mutations, down regulation of E-cadherin expression can also occur via other mechanisms, such as overexpression of transcription repressor, alterations of microRNAs (miRNAs), protein trafficking deregulation, and post-translational modification of the protein [30,31,32]. Recently, glycosylation of E-cadherin has been suggested as another post-translational modification mechanism for its deregulation in many pathophysiological steps of tumor development and progression [33]. The alterations mediated by promoter hyper-methylation and epigenetic inactivation of *CDH1* has been found most commonly in DGC, playing a vital role as a second-hit mechanism in deregulation of the wild-type of *CDH1* in HDGC patients [34,67]. In a recent study, the substitution in *CDH1* encoding for the extracellular domain, such as NM_004360.3: c.2076T > C rs:1801552 in exon 13 together with c.348G > A as a new variant, were found to impair its cell adhesion function and contributed to the development of DGC [64]. On the other hand, substitution NM_004360.3: c.2253C > T rs:33964119 located in exon 14, encoding for the cytoplasmic domain of E-cadherin, was also found in DGC [64]. The cytoplasmic domain of E-cadherin binding with β-catenin plays a critical role in the inhibition of nuclear signaling pathways and tumor-suppression function [68]. In prior studies, the frequency of promoter polymorphism at the −160 position (C > A) of *CDH1* was found to be significantly greater in DGC than in the control groups. The three-marker haplotype (−160C > A, 48 + 6T > C, 2076C > T) was found to significantly contribute to DGC, whereas ATC and ACC haplotypes contributed to higher risk of the development of DGC [35,69,70]. Humar et al. also confirmed that the three-marker haplotype (−160C > A, 48 + 6T > C, 2076C > T) was associated with DGC [35]. In a recent study, impairment of E-cadherin expression was reported with a decreased membranous expression in early lesions of DGC [71]. 

**Figure 2:**
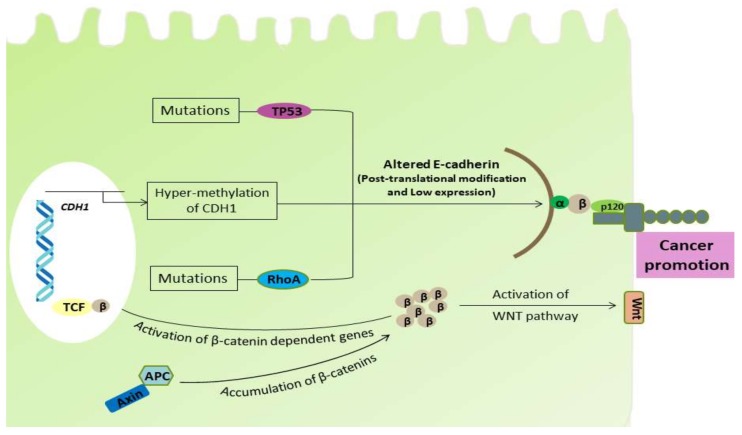
Pathogenicity and factors associated with the disruption of the normal cellular activity. Hyper-methylation of the *CDH1* gene and mutational alteration in TP53 protein causes the impaired synthesis of E-cadherin. The truncated APC causes accumulation of β-catenin, which activates the β-catenin-dependent genes and Wnt pathway, altering normal cellular functions. The Wnt pathway after its activation causes the accumulation of β-catenins in cytoplasm and its translocation into the nucleus where it transcriptionally activates the transcription factors belonging to the TCF family. The recurrent mutation in RhoA is able to alter the RhoA pathway, which has a deleterious effect on E-cadherin.

Park et al. performed a study of gastro-duodenal epithelium-specific knockout of one allele of *CDH1* and both alleles of tumor protein 53 (TP53) and SMAD4 (a homologous gene product to the Caenorhabditis elegans gene (*SMA*) and the Drosophila gene ‘mothers against decapetaplegic’ (*MAD*)), which are the most vulnerable to being inactivated in human GCs. The loss of E-cadherin function together with SMAD4 was found, which underwent epithelium-mesenchymal transition (EMT) and co-operated to promote the development of metastatic progression of TP53-null DGC [72]. This result closely mimicked the human DGC and evaluated the possible role of E-cadherin and SMAD4 in the development of DGC. In addition to its role in cell–cell adhesion, E-cadherin and the cadherin-catenin complex have been demonstrated to modulate various signaling pathways in epithelial cells, such as Wnt signaling, Rho GTPases (a Ras homolog that hydrolyzes the guanosine triphosphate), and nuclear factor kappa-B (NF-κB) pathways [73]. Therefore, impairment of E-cadherin promotes dysfunctions of these signaling pathways, thereby influencing cell polarity, cell survival, invasion and metastasis of gastric cancer, and mainly promotes DGC through the EMT mechanism [74]. Therefore, the cellular events and deregulation in E-cadherin results in the disruption of normal cellular functions (Figure 2).

### 2.2. Alterations in Ras Homolog Gene Family A (RhoA)

Wang et al. conducted a study in 2014 utilizing primary mouse intestinal organoids and determined that the recurrent mutations in RHOA (Y42C and L57V) inhibit the cell death induced when anchorage-dependent cells detach from the surrounding extracellular matrix. This phenomenon is known as anoikis and it plays a key role in the development of DGC [75]. It is well known that the loss of E-cadherin leads to impairment of cellular adhesion, resulting in acute cell death via anoikis. In other words, the alterations or impairment in RHOA function somehow impairs E-cadherin function. Consequently, another study evaluated the role of RHOA mutations associated with DGC [36]. These RHOA mutations in hotspot sites were Y42C, G17E, R5Q/W, and L57V with Y42C being the most common mutation in the effector-binding region of RhoA. In 2014, The Cancer Genome Atlas (TCGA) identified a rate of RHOA mutations in DGC [76]. The TCGA network also found additional fusions in GTPase-activating proteins (GAPs), which are crucial in regulating RhoA activity. More importantly, these mutations were generally found in DGC and not in IGC. Consequently, Ushiku et al. also reported the RHOA mutations causing DGC in 2016 [77]. The impairment of RhoA results in the loss of its expression and activity that may play a role in the development of DGC [78]. RhoA, a member of the Rho family, is a small GTPase protein that plays a fundamental role in regulating diverse cellular processes, such as cell growth, cell survival, polarity, adhesion, cell migration, and differentiation [79,80,81,82]. The studies have shown that genetic alterations in the RhoA pathway, including recurrent RHOA mutations and RhoGAP fusion along with the *CDH1* mutations, are quite common in DGC but not in other variants of gastric cancer [36,76]. These results suggest a possible role of wild-type RhoA in the suppression of DGC development, whereas mutational alterations in RhoA lead to its development, inhibiting the tumor suppression activity (Figure 2).

### 2.3. Role of Sphingosine-1-Phosphate

Sphingosine-1-phosphate (S1P), a bioactive lipid mediator generated by sphingosine kinsase-1 (SphK1) inside the cancer cells, is a key regulatory molecule in cancer via cell proliferation, migration, invasion, and angiogenesis [83,84,85,86,87,88]. S1P, after being generated by cancer cells, is exported to the tumor microenvironment where binding to and signaling through specific G protein-coupled receptors, known as S1PR1-5, regulates many functions [85,86,87,88,89,90,91]. The experimental models conducted by Nagahashi et al. showed that S1P produced by SphK1 in cancer cells promotes lymph node metastasis in tumor microenvironments and promotes lymphangiogenesis [37]. In a recent study, Hanyu et al. reported the role of phosphorylated-SphK1 in the development of DGC and its lymphatic invasion [38].

### 2.4. Role of Adenomatous Polyposis Coli

The gene associated with human adenomatous polyposis coli (APC) is located on the long arm of chromosome 5, which encodes a protein of 312 kDa with 2843 amino acids that acts as a tumor-suppressive protein [92]. A Germline mutation of the APC gene and its inactivation has been found responsible for familial adenomatous polyposis (FAP) [93,94]. Mutations in the APC gene leading to the inactivation of this protein are involved in initiating the carcinogenesis events [92]. The wild-type APC gene product has been found to interact with and degrade β-catenin, whereas truncated APC promotes β-catenin accumulation, activating the members of Wnt signaling pathway that stimulates cell division within intestinal crypts [95]. Therefore, maintenance of low levels of cytosolic β-catenin by functioning APC proteins is essential to prevent excessive cell proliferation [39]. In a recent study by Ghatak et al., the role of somatic mutations in APC (g.127576C > A, g.127583C > T) in exon 14 altering the APC protein expression and cell cycle regulation was shown to contribute to the development of DGC [40].

### 2.5. Role of Fibroblast Growth Factor Receptor (FGFR)

The overexpression of receptor tyrosine kinases (RTKs) has been correlated with the progression and poor survival of GC, whereas the immuno-histochemical overexpression of RTKs variant (i.e., human epidermal growth factor receptor 2—HER2) was found to be associated more frequently in the development of IGC rather than DGC [96,97,98]. The role of genomic alterations in RTKs between IDC and DGC has been revealed in comprehensive genomic analysis performed in TGCA [76]. The fibroblast growth factor receptor (FGFR) family comprises another type of RTKs that interacts with fibroblast growth factors (FGFs) and regulates the essential developmental pathways participating in several biological functions, such as angiogenesis and wound repair [41]. FGFRs also regulate essential cell activities including cell proliferation, survival, migration and differentiation [41]. FGFR2 gene amplification and protein overexpression was found in the GC cell line originating from DGC and it has been recently reported in the development of GCs [99,100]. In a study, the significantly high expression of the FGFR2 protein was commonly reported in DGC rather than IGC [42]. A similar study also showed the significant association of FGFR2 protein overexpression with poor survival and peritoneal dissemination of GC [97]. Moreover, a significant correlation of overexpression of FGFR1 and FGFR2 with tumor progression and survival was found only in DGC, which was also associated with peritoneal dissemination [101]. Therefore, the findings of these studies suggest the possible role of FGFR1 and FGFR2 in DGC development and their association with peritoneal dissemination.

### 2.6. Role of Growth/Differentiation Factor 15 (GDF15)

The results of another study reported the association of growth/differentiating factor 15 (GDF15) with DGC; it was suggested that GDF15 may be the molecules involved in the progression of DGC [102]. Patients with DGC also showed significantly higher serum levels of GDF15, as analyzed by the ELISA method [102]. The secreted growth factors, such as transforming growth factor-β (TGF-β), platelet-derived growth factor (PDGF), and fibroblast growth factor-2 (FGF-2) released by cancer cells, play a key role in the activation of fibroblasts in DGC, and particularly in scirrhous GC [103]. The activated fibroblasts produce various growth factors that help in the progression of scirrhous GC and the secreted proteins play a major role in the molecular pathology of DGC progression [103].

### 2.7. Li-Fraumeni Syndrome with Germline Mutations in Tumor Protein 53 (TP53)

Li-Fraumeni syndrome is genetically inherited in an autosomal dominant manner that is characterized by an accumulation of brain tumors, sarcomas, and breast cancer. Li-Fraumeni syndrome is caused by an alteration in *TP53* (tumor protein 53 or p53), which is a tumor suppressive gene [104]. Upon activation under the cellular stress, the p53 protein performs several functions such as induction of cell cycle arrest and apoptosis, inhibition of cell growth, and interaction with DNA repair proteins [43]. The mutational alteration in *TP53* is considered one of the most prevalent genetic alterations in GC. However, the association of *TP53* mutation with histological-type CG is conflicting [105]. The truncating germline *TP53* mutation was reported in a family characterized by having both HDGC and Li-Fraumeni syndrome [106]. Several other studies highlighted a significant association of the TP53 mutations with the development of DGC rather than IGC [44,45]. The frequent mutations at *TP53*, CKLF-like MARVEL transmembrane domain-containing protein-2 (*CMTM2), CDH1*, and *RHOA* were reported in DGC [46].

### 2.8. Role of Alteration in Other Genes

In addition to the alterations in the E-cadherin gene (*CDH1*), the mutations in the catenin alpha-1 (*CTNNA1*), breast cancer gene (*BRCA2*), serine/threonine kinase-11 (*STK11*), succinate dehydrogenase subunit-B (*SDHB*), serine protease-1 (*PRSS1*), ataxia-telangiectasia mutated gene (*ATM*), macrophage scavenger receptor-1 (*MSR1*), and partner and localizer of BRCA2 (*PALB2*) genes have been reported in the development of DGC [107]. In a recent study, the high frequency mutations for DGC were also reported in lysine methyl-transferase-2D gene (*KMT2D*), AT-rich interactive domain-containing protein 1A (*ARID1A*), *APC*, and phosphatidylinositol 3-kinase catalytic subunit (*PIK3CA*), in addition to high frequency mutations in *TP53*, *CDH1*, and *RHOA* [108]. Alterations in new candidates such as insulin receptor gene (*INSR*), F-box only protein 24 (*FBXO24*), and dot1-like histone lysine methyltransferase (*DOT1L*) have also been reported for DGC susceptibility [109]. Choi et al. found a novel mutation at *CMTM2* in addition to the previously known mutations and they suggested that it may play a crucial role in development of DGC [46]. CMTM2 is a chemokine-like factor that regulates vesicular transport or membrane apposition events belonging to the CMTM family (e.g., CMTM3, CMTM4, CMTM7, and CMTM8), which play a role in the tumor suppression [110,111,112,113].

## 3. *Helicobacter pylori* Infection and DGC

*H. pylori* colonizes the gastric epithelium and persists for several decades. Chronic infections have been found to cause chronic gastritis and atrophic gastritis, a precancerous lesion of gastric cancer. Based on the strong linking evidence of this bacterium with the development of peptic ulcers and gastric cancer, the International Agency for Research on Cancer (IARC) categorized this bacterium as a group I carcinogen (strong carcinogen) in 1994 [114,115]. *H. pylori* infection was initially believed to be associated with the development of IGC, which arises from chronic gastritis, atrophic gastritis, intestinal metaplasia, and dysplasia, whereas the sequence of events for DGC is poorly understood, though it is thought that at least a subset of DGC is due to genetic abnormalities [19,116]. However, unlike HDGC, *H. pylori* and/or Epstein-Barr virus (EBV) infections have been reported to play an essential role in the development of sporadic DGC [117,118,119]. Several other studies have also reported the association of *H. pylori* infection with the development of DGC [120,121,122]. A recent study reported that patients with current infections were prone to developing DGC compared to patients with past infections [123,124]. Similarly, the association of *H. pylori* was found in 85.36% of DGC [125]. There appears to be little difference in the sero-prevalence of *H. pylori* between the two types of cancers, even after adjusting for age. Serological studies confirmed that *H. pylori* infection is associated with both histological types of GC. The studies suggested that patients with a low *H. pylori*-IgG titer are more prone to developing IGC, whereas those with high *H. pylori*-IgG titer are at high risk for developing DGC [18,122,126]. The progression of gastric mucosal atrophy associated with a decrease in *H. pylori* titer may be attributable to the association between past infection or low *H. pylori*-IgG titer and IGC [123]. Gong et al. also reported the association of high *H. pylori*-IgG titer with the development of DGC [124].

*H. pylori* infection has been reported to inhibit several factors responsible for cell–cell adhesion and DGC pathogenicity. Yang et al. demonstrated the cleavage of E-cadherin by *H. pylori* strains SS1 and 26695, producing cytoplasmic fragments to induce apoptosis. Strain SS1 was found to cleave E-cadherin more efficiently at 12 hour and 24 hour [127]. After translocation into the gastric epithelium, the non-phosphorylated CagA binding with E-cadherin results in the separation of E-cadherin and β-catenin complex, which causes accumulation of β-catenin in the cytoplasm and nucleus, which ultimately trans-activates the β-catenin-dependent gene involved in cancer progression [47]. The aberrant activation of β-catenin disrupts the normal apical-junctional complexes, which lead to the loss of cellular polarity [128]. The binding of CagA with E-cadherin results in its down-regulation, together with decreased expression of p120 and aberrant localization from membrane to cytoplasm, which interacts with Rho GTPases and promotes motility and metastasis [129]. Moreover, the unusual localization of p120 to the nucleus, preventing transcriptional repression of the matrix metalloproteinase-7 (*mmp7*) gene, is involved in gastric carcinogenesis [130]. In a recent study, *H. pylori* infection was found to degrade the membrane-bound β-catenin [131]. *H. pylori* infection has been also shown to cause TP53 mutation and a decreased p27 protein expression [48,49]. Non-phosphorylated CagA, in addition to E-cadherin, have been shown to target the phospholipase C-γ, the adaptor protein Grb2, the hepatocyte growth factor receptor c-Met, and other components, leading to the proinflammatory and mitogenic responses that disrupts cell–cell adhesion, cell polarity, and other cellular physiology [132].

Impairment of myelocytomatosis oncogene (*MYC*) expression occurs in a broad range of human cancers, indicating a crucial role in tumor progression [133,134]. The *MYC* gene, located on chromosome 8q24, encoding a transcriptional factor, plays a key role in the regulation of cell cycle progression, growth, proliferation and apoptosis [135,136]. The results of a study indicated that the MYC protein plays a key role in association with *H. pylori* for diffuse type gastric carcinogenesis, whereas it was concluded that the MYC protein is not associated with the tumorigenic pathway of IGC [137].

**Figure 3:**
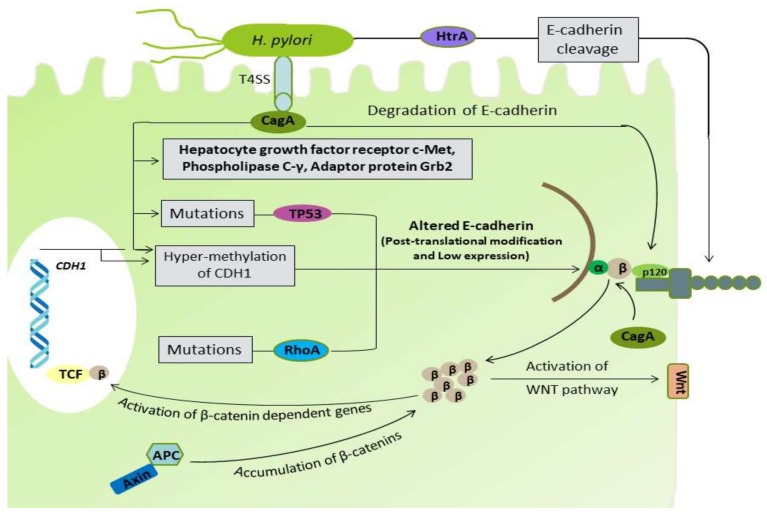
*H. pylori* CagA has an inducible effect on the CDH1 methylation and TP53 mutational alteration. CagA can directly degrade the β-catenin from the E-cadherin-catenins complex. CagA can also degrade the E-cadherin directly. Bacterial HtrA protein can cleave the extracellular domain of E-cadherin.

Aberrant DNA hypermethylation and inactivation of the *CDH1* gene have been found in DGC [34,67]. *H. pylori* infection can induce aberrant hypermethylation of multiple genes, including *CDH1*, leading to the reduction in E-cadherin expression in gastric mucosa, which increases the risk for DGC [50,51]. *H. pylori* serine protease high temperature requirement A (HtrA) is a highly active protein under extreme conditions and degrades the miss-folded protein in bacterial periplasm that enhances the bacterial survival in adverse conditions [138]. In an in vitro infection experiment, the HtrA protein was shown to cleave the extracellular domain of E-cadherin, which led to the opening of the cell junctions in polarized cell monolayers [52]. The results of several other studies identified the *H. pylori* HtrA protein as an E-cadherin targeting protease that directly cleaves-off the extracellular domain of E-cadherin disrupting cell–cell adhesion, leading to cancer development [53,54,55,56]. Moreover, a study conducted by Abdi et al. reported the *H. pylori vacA* d1 type as a potent bacterial virulence factor significantly associated with the development of DGC [139]. Therefore, *H. pylori* proteins, such as CagA, VacA, and HtrA, have regulatory effects on many cellular pathways, and in addition to their role in IGC, they also contribute to the development and prognosis of DGC (Figure 3).

## 4. Hereditary Diffuse Gastric Cancer (HDGC) and Germline Mutations

Although the majority of the DGC cases are sporadic, approximately 1–3% of cases are characterized by inherited syndrome, known as hereditary DGC (HDGC)—an autosomal-dominant cancer susceptibility syndrome characterized by signet ring cell (diffuse) gastric cancer [21,22]. In DGC, the somatic mutations of E-cadherin are described in up to 40–70% of cases, whereas the germline mutations of E-cadherin (*CDH1*), causing loss of its function, are the only proven cause of HDGC, found in approximately 40% of cases [23,140,141]. In 1994, Becker et al. first reported evidence of an inherited form of DGC associated with E-cadherin mutations in specimens from sporadic DGC [113]. In 1998, Guilford et al. found multiple cases of early-onset DGC in Maori ethnic peoples of New Zealand that were carriers of a three germline truncating mutation in the E-cadherin (*CDH1*) gene [23]. Several other publications emerged confirming the association of autosomal-dominant pattern of inheritance with germline mutations of the *CDH1* gene in the following years [26,71,90,107,140,141,142,143,144,145]. Sporadic DGC has shown germline mutations for *CDH1* in a hot spot region between exons 7 and 9, whereas genetic alterations scattered over the entire gene length have been observed for HDGC [146]. Individuals with germline *CDH1* mutations have a single functional *CDH1* allele, whereas the germline *CDH1* alterations in the entire coding region of the other allele may contain small frameshifts, splice-site, nonsense, and missense mutations, as well as large rearrangements. The mutations causing the truncating or pre-matured types are pathogenic, whereas several missense mutations cause impairment of E-cadherin function [147]. Moreover, germline *CDH1* mutations resulting in the complete loss of E-cadherin expression is observed in about 80% of the cases due to the occurrence of premature stop codons causing truncating or non-functional E-cadherin [148,149]. Also, missense-type mutations substituting a single amino acid resulted in full-length E-cadherin in the remaining 20% of HDGC cases [147,148,150,151].

The second hit molecular mechanism causing the inactivation of the remaining functional allele by promoter hyper-methylation was demonstrated to be the most frequent cause of a second hit that leads to the inactivation of both alleles of the E-cadherin (*CDH1*) gene, which is the trigger event for the development of DGC [31,152,153,154]. The second mutation or deletion is an apparently less frequent cause of second hit molecular inactivation of E-cadherin [31,153].

The International Gastric Cancer Linkage Consortium has defined the well-characterized criteria for ruling out HDGC: two GC cases in a family with one individual with confirmed DGC at any age; or three confirmed cases in a family with GC in first- or second-degree relatives regardless of age; or a single case of GC before 40 years of age; or a family history of GC and lobular breast cancer, one diagnosed before 50 years of age [26]. In families meeting the consortium criteria for HDGC carrying the germline mutations are predisposed to an extreme risk of developing DGC from a relatively young age. Based on the familial trace-out of HDGC cases from around the world, the estimated cumulative risk of developing DGC by the age of 80 years has been documented to be 70% for men (95% confidence interval 59–80%) and 56% for women (95% confidence interval 44–69%) [155]. In addition to the risk for DGC, women carrying *CDH1* mutations also possess a cumulative risk of 42% for developing breast cancer, typically the lobular type [155]. However, mutations in *CDH1* are not always associated with the development of GC. In another study, a *CDH1* pathogenic mutation was recorded in a patient but no history of DGC was found in three generations of that family [156]. Similarly, in another study, there was *CDH1* germline missense mutation without any reported history of DGC [157]. Moreover, approximately 60 to 70% of families that fulfill the current testing criteria for HDGC do not possess the germline *CDH1* mutations [152,155]. There has been a few, rare, and highly penetrant familial GC genes; several other familial cancer syndromes also exist for which the GC has a low penetrance feature [158]. Moreover, only about 40% of the probands meeting the 2010 consortium criteria carry germline *CDH1* alterations [159,160]; of the remaining 60%, a small percentage is due to *CDH1* deletions not detected by conventional DNA sequencing and others have shown mutations in other genes such as *CTNNA1* [161], *MAP3K6* [162], *INSR*, *FBXO24*, and *DOT1L* [109]. Hansford et al. showed results from targeted sequencing of 55 cancer-associated genes in 144 families with HDGC who did not possess the detectable germline *CDH1* mutations [155]. They identified two families with germline mutations in *CTNNA1* as well as germline mutations causing truncated type of BRCA2, PRSS1, ATM, PALB2, SDHB, STK11, and MSR1 [155]. 

*CTNNA1* encodes α-catenin, forming a complex with β-catenin to bind the cytoplasmic domain of E-cadherin to the cytoskeleton, is involved in cell–cell adhesion [152]. In a recent study, the comparison of caudal type homeobox-2 protein (CDX2) association with sporadic or HDGC showed that all HDGC cases were negative for CDX2, whereas 19 out of 20 sporadic DGC cases showed CDX2 expression, indicating that sporadic and HDGC may arise via different molecular carcinogenic pathways [163]. Other germline mutations described for familial DGC are in mitogene-activated protein kinase kinase kinase 6 (MAP3K6) and myeloid differentiation primary response protein 88 (MYD88), but their significance in causing DGC is not yet known [162,164]. In summary, germline *CDH1* mutation—however not limited—is frequently associated with the development of HDGC, whereas the mutations in *TP53* and *RHOA*, in addition to the *CDH1* mutations, are documented in sporadic-type DGC. However, the detailed molecular mechanisms underlying the development of DGC have not yet been clarified in detail [36,46,76].

## 5. Conclusions

Although the detailed pathogenicity of DGC is not well described, the combined information presented in this report indicates that development of DGC involves multiple factors of cell signaling pathways, cell–cell adhesion, and *H. pylori* infection. The E-cadherin and cell-signaling pathways play a vital role in the maintenance of cell integrity and normal cell function. Deregulation and alterations in these molecules disrupt the normal cellular functions that contribute to the initiation and progression of gastric cancer. The alterations in E-cadherin have been known as a factor strongly associated factor with DGC, with other less frequently associated and newly identified factors. Despite its role in IDC, *H. pylori* has been found to influence the development of DGC. However, more details and further investigations are needed.

## Figures and Tables

**Table 1:** Contributing factors for pathogenicity of diffuse type gastric cancer (DGC).

Factor	Mechanism	Effects	References
Host Factor			
E-cadherin (*CDH1*)	Mutational alterations	Deregulation of E-cadherin	[28,29]
	Over expression of transcription repressor	Down regulation of E-cadherin	[30,31,32]
	Post-translational modification	Glycosylation modification of E-cadherin	[33]
	Promoter hyper-methylation	E-cadherin inactivation	[34]
	Promoter polymorphism	Alterations in E-cadherin	[35]
Ras homolog gene family A (RHOA)	Mutational alterations	Loss of E-cadherin activity	[36]
Sphingosine-1-phosphate (S1P)	Synthesis	Development of DGC and lymphatic invasion	[37,38]
Adenomatous polyposis coli (APC)	Mutations leading to altered expression of APC protein	Accumulation of β-catenin leading to the activation of Wnt-signaling pathway	[39,40]
Fibroblast growth factor receptor (FGFR2)	Overexpression	Inhibition in the cellular activities	[41,42]
Tumor protein 53 (TP53)	Mutational alteration	Loss of cell regulating mechanism	[43,44,45,46]
***Helicobacter pylori***			
Non-phosphorylated CagA	Binds with E-cadherin	Dissociation of E-cadherin-β-catenin complex	[47]
	Causes mutational alterations in TP53	Impairment of E-cadherin synthesis	[48,49]
	Causes hyper-methylation of *CDH1*	Reduced E-cadherin expression	[50,51]
High temperature requirement A (HtrA)	Causes cleavage of extracellular domain of E-cadherin	Disruption of normal cell junctions	[52,53,54,55,56]
